# Crystal structure of a new homochiral one-dimensional zincophosphate containing l-me­thio­nine

**DOI:** 10.1107/S2056989015011561

**Published:** 2015-06-24

**Authors:** Nadjet Chouat, Mohammed Abdelkrim Hasnaoui, Mohamed Sassi, Abdelkader Bengueddach, Gigliola Lusvardi, Andrea Cornia

**Affiliations:** a Laboratoire de Chimie des Matériaux, Oran University, BP 1524, El M’nouar, 31000 Oran, Algeria; b Dipartimento di Scienze Chimiche e Geologiche, University of Modena and Reggio Emilia, and INSTM RU, via G. Campi 103, 41125, Modena, Italy

**Keywords:** crystal structure, hydro­thermal synthesis, zincophosphates, me­thio­nine, hybrid materials, homochiral structure

## Abstract

*catena*-Poly[[(l-me­thio­nine-κ*O*)zinc]-μ_3_-(hydrogen phosphato)-κ^3^ *O*:*O*′:*O*′′], [Zn{PO_3_(OH)}(C_5_H_11_NO_2_S)]_*n*_, a new one-dimensional homochiral zincophos­phate, was hydro­thermally synthesized using l-me­thio­nine as a structure-directing agent. The compound consists of a network of ZnO_4_ and (HO)PO_3_ tetra­hedra that form ladder-like chains of edge-fused Zn_2_P_2_O_4_ rings propagating parallel to [100]. The chains are decorated on each side by zwitterionic l-me­thio­nine ligands, which inter­act with the inorganic framework *via* Zn—O coordination bonds. The structure displays inter­chain N—H⋯O and O—H⋯S hydrogen bonds.

## Abstract 2

[Zn(HPO_4_)(l-met)]_*n*_ is a rare example of a one-dimensional zincophosphate compound with a homochiral structure. Ladder-like chains of alternating ZnO_4_ and (HO)PO_3_ tetra­hedra are bedecked on each side by zwitterionic l-me­thio­nine ligands, which inter­act with the inorganic framework *via* Zn—O coordination bonds.

## Chemical context   

In the last two decades, the blossoming of research on hybrid organic-inorganic open-framework systems has been motivated by the growing inter­est in obtaining materials that combine the functional properties of organic and inorganic components (Wang *et al.*, 2014 ▸; Murugavel *et al.*, 2008 ▸; Thomas, 1994 ▸). Since their discovery in 1991 (Gier & Stucky, 1991 ▸), attention on hybrid zincophosphates has arisen because of the diversity of new open-framework structures that can be obtained (Kefi *et al.*, 2007 ▸; Fleith *et al.*, 2002 ▸; Stojakovic *et al.*, 2009 ▸; Mekhatria *et al.*, 2011 ▸). Although in the majority of cases the organic mol­ecules are hydrogen-bonded to the mineral framework or trapped in the micropores of the material, they can also be directly linked to the inorganic network through coordination bonds (Mekhatria *et al.*, 2011 ▸; Fan *et al.*, 2005 ▸; Fan & Hanson, 2005 ▸; Zhao *et al.*, 2008 ▸; Dong *et al.*, 2010 ▸). In such systems and in the related class of zincophosphites, amino acids have been used as chiral structure-directing agents with only partial success. Enanti­opure histidine, for example, has been shown to template the formation of zincophosphate (Mekhatria *et al.*, 2011 ▸; Fan *et al.*, 2005 ▸; Zhao *et al.*, 2008 ▸) or zincophosphite (Chen & Bu, 2006 ▸) materials. The amino acid coordinates the Zn atom *via* either its carboxyl­ate group (Mekhatria *et al.*, 2011 ▸; Zhao *et al.*, 2008 ▸), its imidazole ring (Fan *et al.*, 2005 ▸) or both functions (Chen & Bu, 2006 ▸). However, racemization of histidine takes place during the synthesis and the reported materials are achiral. Among the rare homochiral systems so far assembled are ladder-like zincophosphites [HA·ZnHPO_3_] where the amino­acid [HA = l-asparagine (Gordon & Harrison, 2004 ▸) or l-tryptophan (Dong *et al.*, 2010 ▸)] is O-bound to the inorganic framework. Using l-histidine, a zincophosphate [Zn_3_(H_2_O)(PO_4_)(HPO_4_)(HA)_2_(A)] was also isolated displaying ladder-like chains decorated by pendant ZnO_2_N_2_ tetra­hedra (Dong *et al.*, 2010 ▸). In this material, the two neutral amino acid mol­ecules act as monodentate ligands through their imidazole function, while the deprotonated one chelates a Zn atom *via* its imidazole and amino groups.
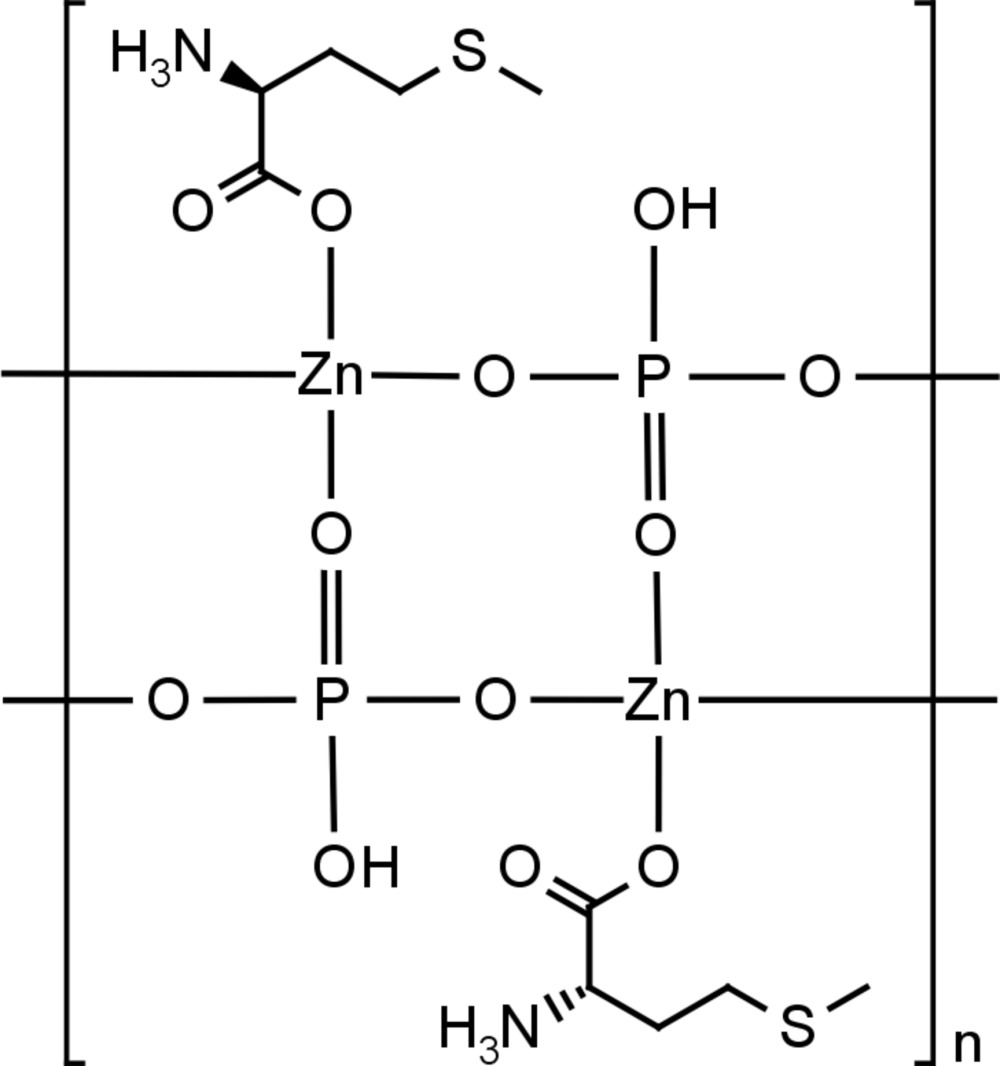


We report herein a new zincophosphate compound, [Zn(HPO_4_)(l-met)]_*n*_ (I), containing O-bound l-me­thio­nine (l-met) and exhibiting a simple ladder-like homochiral structure. The compound was obtained as a minority phase together with hopeite [Zn_3_(PO_4_)_2_·4H_2_O; Hill & Jones, 1976 ▸] and residues of the reagents by hydro­thermal synthesis starting from zinc oxide, ortho­phospho­ric acid and l-me­thio­nine in water. A needle-like single crystal of sufficient size and quality was isolated from the product mixture and a single-crystal X-ray analysis performed at room temperature.

## Structural commentary   

**Figure 1:**
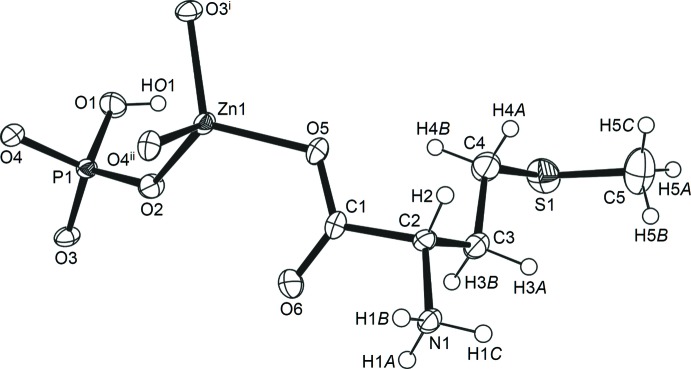
The asymmetric unit of (I), plus the O atoms required to complete the coordination sphere of Zn. Displacement ellipsoids are drawn at the 40% probability level, while H atoms are shown as spheres of arbitrary radius. [Symmetry codes: (i) *x* − 1, *y*, *z*; (ii) *x* − 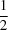, 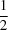 − *y*, 1 − *z*].

The asymmetric unit contains one zinc cation, one hydrogenphosphate anion and one l-me­thio­nine ligand in its zwitterionic form. It is shown in Fig. 1 ▸ along with the symmetry-equivalent O atoms required to complete the coordination sphere of Zn. Such a formulation is in accordance with charge balance considerations assuming usual valences for Zn (2+), P (5+), O (2−) and H (1+). The ammonium and HPO_4_^2−^ hydrogen atoms were clearly located in Fourier difference maps. The zinc ion is tetra­hedrally coordinated by the oxygen atoms (O2, O3^i^ and O4^ii^) of three different (HO)PO_3_^2−^ groups and by the carboxyl­ate oxygen (O5) of me­thio­nine, with (Zn—O)_av_ = 1.95 Å and O—Zn—O angles in the range 103.84 (11)–115.56 (11)° (Table 1 ▸). The hydrogenphosphate group is connected to three different zinc ions through O2, O3 and O4. The corresponding P—O distances range between 1.510 (3) and 1.525 (2) Å while the terminal P1—O1 bond is much longer [1.584 (3) Å], as expected for a pendant OH group (Fan *et al.*, 2005 ▸; Fan & Hanson, 2005 ▸). The O—P—O and Zn—O—P angles are in the ranges 103.27 (14)–114.41 (14) and 129.16 (14)–132.83 (15)°, respectively.

**Figure 2:**
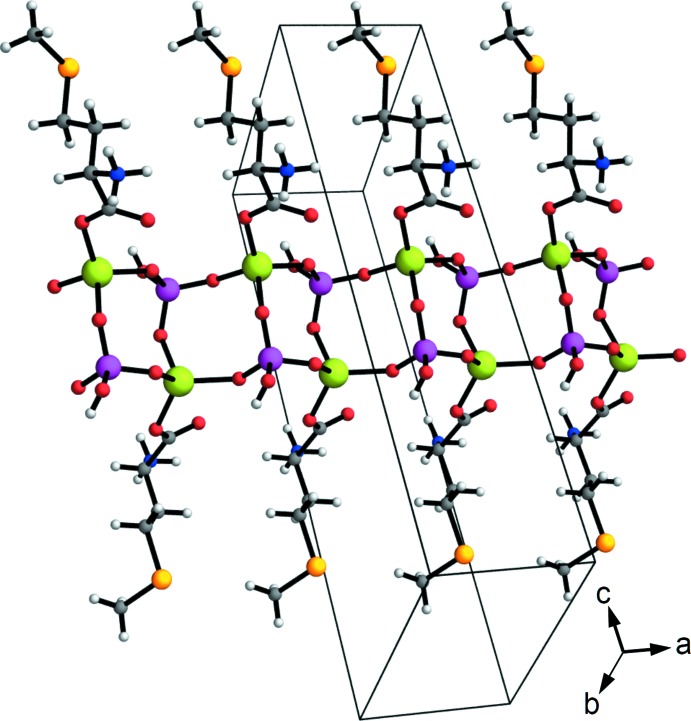
Ladder-like chains running parallel to [100] and decorated by l-me­thio­nine ligands in the structure of (I). Atoms are depicted as spheres with arbitrary radius. Color code: C gray, N blue, O red, H light gray, P purple, Zn green.

As a consequence of the 2_1_ axis lying parallel to [100], the alternating ZnO_4_ and (HO)PO_3_ tetrahedra form neutral ladder-like chains of edge-fused Zn_2_P_2_O_4_ rings that propagate parallel to the [100] direction (Fig. 2 ▸). l-Me­thio­nine mol­ecules are grafted on each side of the ladder and act as monodentate ligands rather than as a chelants (Brand *et al.*, 2001 ▸). The geometrical parameters of the amino acid are unexceptional for zwitterionic me­thio­nine (Alagar *et al.*, 2005 ▸). No extra framework components are present. As its most inter­esting aspect, the structure is homochiral: all me­th­io­nine ancillary ligands have the same *S* configuration at their C2 atoms as in the starting material (l-me­thio­nine). Such a structure is similar to that previously reported for zincophosphite chains (Dong *et al.*, 2010 ▸; Gordon & Harrison, 2004 ▸) but is, to the best of our knowledge, unknown for zinco­phos­phates.

## Supra­molecular features   

**Figure 3:**
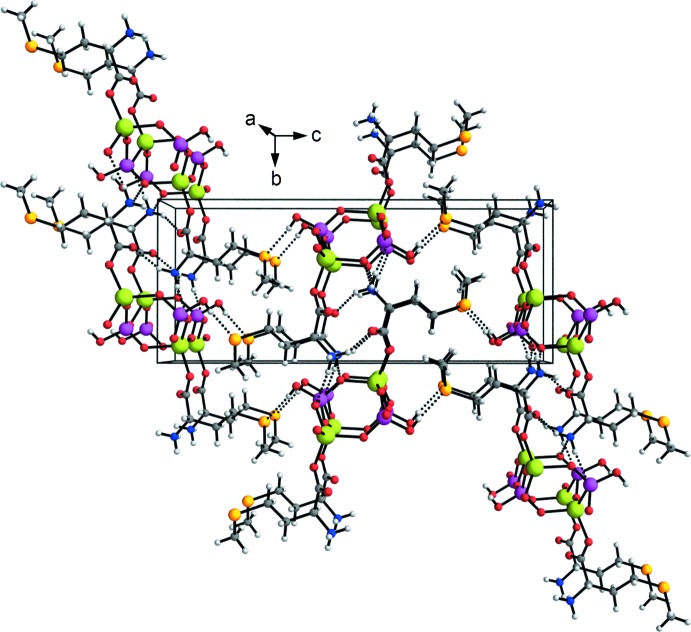
Crystal packing diagram for compound (I), viewed along [100]. Dashed lines represent hydrogen-bonding inter­actions (see Table 2 ▸ for details). Atoms are depicted as spheres with arbitrary radius using the same color code as in Fig. 2 ▸.

No intra­chain hydrogen bonds are present, differing from the l-asparagine derivative described by Gordon & Harrison (2004 ▸). The ladder-like chains in (I) are assembled *via* a network of hydrogen-bonding inter­actions (Fig. 3 ▸ and Table 2 ▸). The ammonium group is engaged in three hydrogen bonds with a neighboring chain obtained by unitary translation along [010]. The hydrogen-bond acceptors are the HPO_4_^2−^ oxygen atoms O3 and O4 and the non-coordinating carboxyl­ate oxygen O6 of the me­thio­nine ligand. Along the [001] direction, the ladders are linked by hydrogen bonds between the pendant OH groups and the me­thio­nine sulfur atoms.

## Synthesis and crystallization   

The reaction mixture, with a molar composition of 2:1:1:180 for ZnO:P_2_O_5_:l-me­thio­nine:H_2_O, was prepared by mixing zinc oxide (Merck, 99%) with an appropriate amount of distilled water. Proper amounts of ortho­phospho­ric acid (Biochem, 98%) and l-me­thio­nine (Merck, 99%) were then added, under stirring. After heating at 373 K for 3 days, the solid obtained was recovered, washed with distilled water and dried at 333 K overnight. The solid product, consisting of small shiny crystals, turned out to be multiphasic, with hopeite and (I) as major components. Qualitative and qu­anti­tative phase analyses by powder XRD and Rietveld refinement gave (wt%): 80±1% of hopeite, 7.0±0.5% of (I), 2±0.2% of l-me­thio­nine, 1±0.2% of zinc oxide and 10±1% of an amorphous phase. Such a composition is in reasonable agreement with the C, H, N, S content of the bulk phase determined by combustion analysis. Analysis calculated (wt%) for the composition resulting from Rietveld refinement (neglecting the amorphous phase): C, 2.16 (13); H, 1.83 (3); N, 0.50 (3); S, 1.15 (7). Found: C, 2.5; H, 1.9; N, 0.6; S, 2.4. The occurrence of hopeite and (I) as main phases was confirmed by scanning electron microscopy and semi-qu­anti­tative EDS analysis. So far, we have been unable to isolate the new compound in pure form, and attempts to crystallize it in fluoride medium remained unsuccessful.

## Refinement   

Crystal data, data collection and structure refinement details are summarized in Table 3 ▸. C-bound H atoms were added in calculated positions with C—H = 0.98, 0.97, 0.96 Å for tertiary, secondary and methyl hydrogen atoms, respectively (the CH_3_ group was subjected to torsion-angle refinement). Isotropic displacement parameters for C—H hydrogen atoms were constrained to those of the parent atom, with *U*_iso_(H) = 1.5*U*_eq_(C) for methyl and *U*_iso_(H) = 1.2*U*_eq_(C) for the remaining hydrogen atoms. In a subsequent Δ*F* map, four electron-density residuals were clearly located close to the nitro­gen atom and to the non-bridging phosphate oxygen atom and refined as the ammonium and hydrogenphosphate H atoms, respectively. The ammonium group was constrained to have an idealized geometry with N—H = 0.89 Å and was subjected to torsion-angle refinement with a common *U*_iso_ value for its H atoms. Note that when the occupancy factor of N-bound hydrogen atoms was decreased to 2/3, to model a rotationally disordered amino group, their *U*_iso_ refined to an unphysically low value. The hydroxyl hydrogen atom was refined freely, but the O—H distance was restrained to 0.82 (1) Å. The Flack parameter for the complete structural model was *x* = 0.054 (16) by a classical fit to all intensities (Flack, 1983 ▸) and 0.063 (10) from 841 selected quotients (Parsons *et al.*, 2013 ▸). The final refinement was then carried out as a two-component inversion twin, resulting in a 0.055 (16) fraction of the inverted component.

## Supplementary Material

Crystal structure: contains datablock(s) I, global. DOI: 10.1107/S2056989015011561/wm5165sup1.cif

Structure factors: contains datablock(s) I. DOI: 10.1107/S2056989015011561/wm5165Isup2.hkl

Supporting information file. DOI: 10.1107/S2056989015011561/wm5165Isup3.pdf

CCDC reference: 1012270

Additional supporting information:  crystallographic information; 3D view; checkCIF report

## Figures and Tables

**Table 1:** Selected bond lengths ()

Zn1O2	1.936(2)	P1O1	1.584(3)
Zn1O3^i^	1.940(2)	P1O2	1.510(3)
Zn1O4^ii^	1.968(2)	P1O3	1.525(2)
Zn1O5	1.943(3)	P1O4	1.522(2)

Symmetry codes: (i) 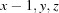; (ii) 
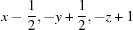
.

**Table 2:** Hydrogen-bond geometry (, )

*D*H*A*	*D*H	H*A*	*D* *A*	*D*H*A*
O1H*O*1S1^iii^	0.81(1)	2.37(1)	3.177(3)	175(5)
N1H1*A*O4^iv^	0.89	2.07	2.820(3)	141
N1H1*B*O6^v^	0.89	1.99	2.785(4)	149
N1H1*C*O3^vi^	0.89	2.05	2.931(4)	172

Symmetry codes: (iii) 
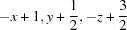
; (iv) 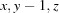; (v) 
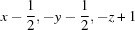
; (vi) 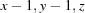.

**Table 3:** Experimental details

Crystal data	
Chemical formula	[Zn(HPO_4_)(C_5_H_11_NO_2_S)]
*M*_r_	310.56
Crystal system, space group	Orthorhombic, *P*2_1_2_1_2_1_
Temperature (K)	298
*a*, *b*, *c* ()	5.2210(2), 9.1889(4), 22.1559(10)
*V* (^3^)	1062.93(8)
*Z*	4
Radiation type	Mo *K*
(mm^1^)	2.67
Crystal size (mm)	0.33 0.07 0.01

Data collection	
Diffractometer	BrukerNonius X8 APEX four-circle
Absorption correction	Multi-scan (*SADABS*; Bruker, 2008 ▸)
*T*_min_, *T*_max_	0.804, 0.974
No. of measured, independent and observed [*I* > 2(*I*)] reflections	7417, 2699, 2334
*R*_int_	0.029
(sin /)_max_ (^1^)	0.682

Refinement	
*R*[*F* ^2^ > 2(*F* ^2^)], *wR*(*F* ^2^), *S*	0.026, 0.056, 1.00
No. of reflections	2699
No. of parameters	144
No. of restraints	1
H-atom treatment	H atoms treated by a mixture of independent and constrained refinement
_max_, _min_ (e ^3^)	0.39, 0.36
Absolute structure	Refined as an inversion twin
Absolute structure parameter	0.055(16)

Computer programs: *APEX2* and *SAINT* (Bruker, 2008 ▸), *SIR92* (Altomare *et al.*, 1993 ▸), *SHELXL2014* (Sheldrick, 2015 ▸) and *ORTEP-3 for Windows* and *WinGX* (Farrugia, 2012 ▸).

## supplementary crystallographic information

### Crystal data

**Table:**

[Zn(HPO_4_)(C_5_H_11_NO_2_S)]	*D*_x_ = 1.941 Mg m^−^^3^
*M**_r_* = 310.56	Mo *K*α radiation, λ = 0.71073 Å
Orthorhombic, *P*2_1_2_1_2_1_	Cell parameters from 2621 reflections
*a* = 5.2210 (2) Å	θ = 2.4–28.2°
*b* = 9.1889 (4) Å	µ = 2.67 mm^−^^1^
*c* = 22.1559 (10) Å	*T* = 298 K
*V* = 1062.93 (8) Å^3^	Needle, colourless
*Z* = 4	0.33 × 0.07 × 0.01 mm
*F*(000) = 632	

### Data collection

**Table:**

Bruker–Nonius X8 APEX four-circle diffractometer	2699 independent reflections
Radiation source: fine-focus sealed tube	2334 reflections with *I* > 2σ(*I*)
Graphite monochromator	*R*_int_ = 0.029
Detector resolution: 66 pixels mm^-1^	θ_max_ = 29.0°, θ_min_ = 2.4°
phi and ω scans	*h* = −6→6
Absorption correction: multi-scan (*SADABS*; Bruker, 2008)	*k* = −8→12
*T*_min_ = 0.804, *T*_max_ = 0.974	*l* = −28→30
7417 measured reflections	

### Refinement

**Table:**

Refinement on *F*^2^	Secondary atom site location: difference Fourier map
Least-squares matrix: full	Hydrogen site location: mixed
*R*[*F*^2^ > 2σ(*F*^2^)] = 0.026	H atoms treated by a mixture of independent and constrained refinement
*wR*(*F*^2^) = 0.056	*w* = 1/[σ^2^(*F*_o_^2^) + (0.0227*P*)^2^] where *P* = (*F*_o_^2^ + 2*F*_c_^2^)/3
*S* = 1.00	(Δ/σ)_max_ = 0.001
2699 reflections	Δρ_max_ = 0.39 e Å^−^^3^
144 parameters	Δρ_min_ = −0.36 e Å^−^^3^
1 restraint	Absolute structure: Refined as an inversion twin
Primary atom site location: structure-invariant direct methods	Absolute structure parameter: 0.055 (16)

### Special details

**Table:**

Geometry. All e.s.d.'s (except the e.s.d. in the dihedral angle between two l.s. planes) are estimated using the full covariance matrix. The cell e.s.d.'s are taken into account individually in the estimation of e.s.d.'s in distances, angles and torsion angles; correlations between e.s.d.'s in cell parameters are only used when they are defined by crystal symmetry. An approximate (isotropic) treatment of cell e.s.d.'s is used for estimating e.s.d.'s involving l.s. planes.
Refinement. After all nonhydrogen atoms were located and refined anisotropically, the model converged to *wR*(*F*^2^) = 0.0877 with a Flack parameter (determined by classical fit to all intensities) *x* = 0.044 (17) (Flack, 1983); for the inverted structure, the same parameters were 0.1288 and 0.94 (3), respectively. The absolute structure was then well defined and corresponded to an *L* configuration for the methionine ligand. C-bound hydrogen atoms were added in calculated positions with C—H = 0.98, 0.97, 0.96 Å for tertiary, secondary and methyl H atoms, respectively (the CH_3_ group was subject to torsion angle refinement using AFIX 137 instruction). Isotropic displacement parameters for C—H H atoms were constrained to those of the parent atom, with *U*_iso_(H) = 1.5*U*_eq_(C) for methyl and *U*_iso_(H) = 1.2*U*_eq_(C) for the remaining H atoms. In a subsequent Δ*F* map, four electron density residuals were clearly located close to the nitrogen atom and to the nonbridging phosphate oxygen and refined as the ammonium and hydrogenphosphate H atoms, respectively. The ammonium group was constrained to have an idealized geometry with N—H = 0.89 Å and was subject to torsion angle refinement with a common *U*_iso_ value for its H atoms. Note that when the occupancy factor of N-bound H atoms was decreased to 2/3, to model a rotationally disordered amino group, their *U*_iso_ refined to an unphysically low value. The hydroxyl hydrogen was refined freely, but the O—H distance was restrained to 0.82 (1) Å. The Flack parameter for the complete structural model was *x* = 0.054 (16) by classical fit to all intensities (Flack, 1983) and 0.063 (10) from 841 selected quotients (Parsons *et al.*, 2013). Final refinement was carried out as a 2-component inversion twin, resulting in a 0.055 (16) fraction of inverted component.

### Fractional atomic coordinates and isotropic or equivalent isotropic displacement parameters (Å^2^)

**Table:**

	*x*	*y*	*z*	*U*_iso_*/*U*_eq_	
Zn1	0.50773 (8)	0.10750 (4)	0.55705 (2)	0.01929 (10)	
P1	1.01002 (19)	0.29242 (8)	0.57930 (3)	0.01729 (16)	
S1	0.3692 (2)	−0.35340 (15)	0.77437 (5)	0.0446 (3)	
O1	0.9167 (5)	0.3698 (3)	0.63929 (12)	0.0322 (6)	
HO1	0.848 (8)	0.309 (4)	0.6596 (19)	0.056 (16)*	
O2	0.8659 (4)	0.1515 (3)	0.57061 (11)	0.0253 (5)	
O3	1.2977 (4)	0.2677 (3)	0.58537 (11)	0.0226 (5)	
O4	0.9594 (4)	0.4063 (3)	0.53087 (10)	0.0236 (5)	
O5	0.3925 (5)	−0.0803 (3)	0.58683 (12)	0.0320 (6)	
O6	0.7309 (5)	−0.1965 (3)	0.54837 (13)	0.0347 (7)	
N1	0.4865 (6)	−0.4489 (3)	0.54404 (11)	0.0213 (5)	
H1A	0.6565	−0.4529	0.5467	0.040 (7)*	
H1B	0.4419	−0.4252	0.5065	0.040 (7)*	
H1C	0.4207	−0.5354	0.5533	0.040 (7)*	
C1	0.5183 (7)	−0.1933 (3)	0.57187 (13)	0.0218 (6)	
C2	0.3873 (6)	−0.3376 (4)	0.58669 (15)	0.0220 (7)	
H2	0.2019	−0.3274	0.5812	0.026*	
C3	0.4427 (7)	−0.3861 (4)	0.65115 (14)	0.0284 (8)	
H3A	0.3895	−0.4866	0.6560	0.034*	
H3B	0.6258	−0.3814	0.6583	0.034*	
C4	0.3057 (8)	−0.2928 (5)	0.69771 (17)	0.0368 (9)	
H4A	0.1227	−0.2962	0.6902	0.044*	
H4B	0.3610	−0.1925	0.6934	0.044*	
C5	0.1420 (9)	−0.4984 (6)	0.7819 (2)	0.0594 (13)	
H5A	0.1428	−0.5333	0.8228	0.089*	
H5B	0.1875	−0.5764	0.7551	0.089*	
H5C	−0.0259	−0.4634	0.7719	0.089*	

### Atomic displacement parameters (Å^2^)

**Table:**

	*U*^11^	*U*^22^	*U*^33^	*U*^12^	*U*^13^	*U*^23^
Zn1	0.01776 (17)	0.01329 (16)	0.02682 (17)	−0.0009 (2)	−0.0005 (2)	0.00083 (13)
P1	0.0160 (4)	0.0145 (4)	0.0214 (3)	0.0000 (5)	0.0011 (4)	−0.0014 (3)
S1	0.0541 (7)	0.0502 (8)	0.0295 (5)	−0.0040 (6)	0.0060 (5)	−0.0076 (5)
O1	0.0388 (16)	0.0272 (16)	0.0306 (13)	0.0009 (11)	0.0122 (11)	−0.0057 (11)
O2	0.0157 (11)	0.0208 (13)	0.0395 (14)	−0.0035 (10)	−0.0027 (10)	−0.0015 (11)
O3	0.0156 (11)	0.0180 (13)	0.0343 (13)	0.0017 (10)	−0.0031 (10)	−0.0025 (10)
O4	0.0252 (14)	0.0208 (12)	0.0248 (10)	0.0049 (12)	0.0017 (9)	0.0036 (9)
O5	0.0373 (14)	0.0133 (13)	0.0456 (15)	−0.0019 (11)	0.0130 (12)	0.0017 (11)
O6	0.0283 (14)	0.0252 (16)	0.0505 (17)	−0.0063 (12)	0.0121 (12)	0.0004 (13)
N1	0.0229 (14)	0.0147 (12)	0.0264 (13)	−0.0005 (16)	−0.0011 (15)	−0.0007 (9)
C1	0.0261 (17)	0.0162 (15)	0.0232 (14)	−0.0036 (19)	−0.0021 (17)	0.0029 (10)
C2	0.0202 (16)	0.0148 (17)	0.0309 (18)	−0.0001 (14)	0.0037 (14)	−0.0037 (14)
C3	0.035 (2)	0.0196 (18)	0.0306 (16)	0.0021 (15)	0.0038 (14)	0.0012 (14)
C4	0.050 (2)	0.029 (2)	0.032 (2)	0.0037 (19)	0.0103 (19)	0.0007 (17)
C5	0.082 (3)	0.052 (3)	0.045 (3)	−0.019 (3)	0.002 (3)	0.007 (2)

### Geometric parameters (Å, º)

**Table:**

Zn1—O2	1.936 (2)	S1—C4	1.818 (4)
Zn1—O3^i^	1.940 (2)	S1—C5	1.792 (5)
Zn1—O4^ii^	1.968 (2)	O1—HO1	0.807 (13)
Zn1—O5	1.943 (3)	N1—H1A	0.8900
P1—O1	1.584 (3)	N1—H1B	0.8900
P1—O2	1.510 (3)	N1—H1C	0.8900
P1—O3	1.525 (2)	C2—H2	0.9800
P1—O4	1.522 (2)	C3—H3A	0.9700
O5—C1	1.272 (4)	C3—H3B	0.9700
O6—C1	1.226 (4)	C4—H4A	0.9700
C1—C2	1.528 (4)	C4—H4B	0.9700
N1—C2	1.486 (4)	C5—H5A	0.9600
C2—C3	1.524 (5)	C5—H5B	0.9600
C3—C4	1.521 (5)	C5—H5C	0.9600
			
O2—Zn1—O3^i^	109.71 (10)	O5—C1—C2	114.9 (3)
O2—Zn1—O5	115.56 (11)	N1—C2—C3	109.2 (3)
O3^i^—Zn1—O5	112.91 (11)	N1—C2—C1	107.8 (3)
O2—Zn1—O4^ii^	106.90 (10)	C3—C2—C1	111.7 (3)
O3^i^—Zn1—O4^ii^	107.25 (10)	N1—C2—H2	109.4
O5—Zn1—O4^ii^	103.84 (11)	C3—C2—H2	109.4
O2—P1—O4	114.41 (14)	C1—C2—H2	109.4
O2—P1—O3	111.98 (14)	C4—C3—C2	112.4 (3)
O4—P1—O3	109.60 (14)	C4—C3—H3A	109.1
O2—P1—O1	109.81 (15)	C2—C3—H3A	109.1
O4—P1—O1	103.27 (14)	C4—C3—H3B	109.1
O3—P1—O1	107.20 (14)	C2—C3—H3B	109.1
C5—S1—C4	101.2 (2)	H3A—C3—H3B	107.9
P1—O1—HO1	107 (4)	C3—C4—S1	112.0 (3)
P1—O2—Zn1	132.83 (15)	C3—C4—H4A	109.2
P1—O3—Zn1^iii^	129.87 (15)	S1—C4—H4A	109.2
P1—O4—Zn1^iv^	129.16 (14)	C3—C4—H4B	109.2
C1—O5—Zn1	118.4 (2)	S1—C4—H4B	109.2
C2—N1—H1A	109.5	H4A—C4—H4B	107.9
C2—N1—H1B	109.5	S1—C5—H5A	109.5
H1A—N1—H1B	109.5	S1—C5—H5B	109.5
C2—N1—H1C	109.5	H5A—C5—H5B	109.5
H1A—N1—H1C	109.5	S1—C5—H5C	109.5
H1B—N1—H1C	109.5	H5A—C5—H5C	109.5
O6—C1—O5	126.7 (3)	H5B—C5—H5C	109.5
O6—C1—C2	118.4 (3)		

Symmetry codes: (i) *x*−1, *y*, *z*; (ii) *x*−1/2, −*y*+1/2, −*z*+1; (iii) *x*+1, *y*, *z*; (iv) *x*+1/2, −*y*+1/2, −*z*+1.

### Hydrogen-bond geometry (Å, º)

**Table:**

*D*—H···*A*	*D*—H	H···*A*	*D*···*A*	*D*—H···*A*
O1—H*O*1···S1^v^	0.81 (1)	2.37 (1)	3.177 (3)	175 (5)
N1—H1*A*···O4^vi^	0.89	2.07	2.820 (3)	141
N1—H1*B*···O6^vii^	0.89	1.99	2.785 (4)	149
N1—H1*C*···O3^viii^	0.89	2.05	2.931 (4)	172

Symmetry codes: (v) −*x*+1, *y*+1/2, −*z*+3/2; (vi) *x*, *y*−1, *z*; (vii) *x*−1/2, −*y*−1/2, −*z*+1; (viii) *x*−1, *y*−1, *z*.
