# Supplementary material for: Crystal structure of a new homochiral one-dimensional zincophosphate containing l-me­thio­nine
Source: Acta Crystallogr E Crystallogr Commun. 2015 Jun 24;71(Pt 7):832–5. doi: 10.1107/S2056989015011561 (PMC4518946; doi:10.1107/S2056989015011561)
Supplement: Supplementary file 3 [file e-71-00832-Isup3.pdf]

Supporting Information

Physical measurements

The powder XRD pattern was recorded on a Bragg-Brentano diffractometer (PANalytical X'Pert Pro, CuK_α radiation, Ni-filtered) over the range 2θ = 3-100° with a counting time of 100 s for each 0.03° step in 2θ. Powders were uniformly hand-ground in an agate mortar before the data collection. Identification [1] of the crystalline phases was followed by Quantitative Phase Analysis (QPA) obtained by means of the Rietveld refinement [2]. The refinement of the spectrum was performed using the GSAS program [3] and its graphical interface EXPGUI [4]. The structural model for (I) was based on the single-crystal X-ray investigation herein reported; for all the other phases data were taken from the ICSD database [5]. An internal standard (10 wt%, α-alumina, NIST SRM 676) was added as for the evaluation of the amorphous phase content.

The following parameters were refined: Chebyshev polynomial background function, zero-shift; for each phase the refined parameters were scale factor, unit-cell parameters, a Gaussian and a Lorentzian coefficient of the pseudo–Voigt peak-profile function, an offset function for the correction of the peak asymmetry and the sample-displacement correction.

Agreement factors as defined in GSAS were: χ^2 ~ 3, R_{wp} (%) ~ 9, $R_F{}^2$ (%) ~ 10.

The morphological and elemental analyses of the solid products were carried out by means of Environmental Scanning Electron Microscopy–Energy Dispersive Spectroscopy (ESEM-EDS) using a FEI Quanta 200 instrument (Fei Company, The Netherlands), equipped with an INCA 350 EDS apparatus (Oxford Instruments, UK); EDS analysis was performed over different areas of the sample and the maximum value of σ was 0.5%.

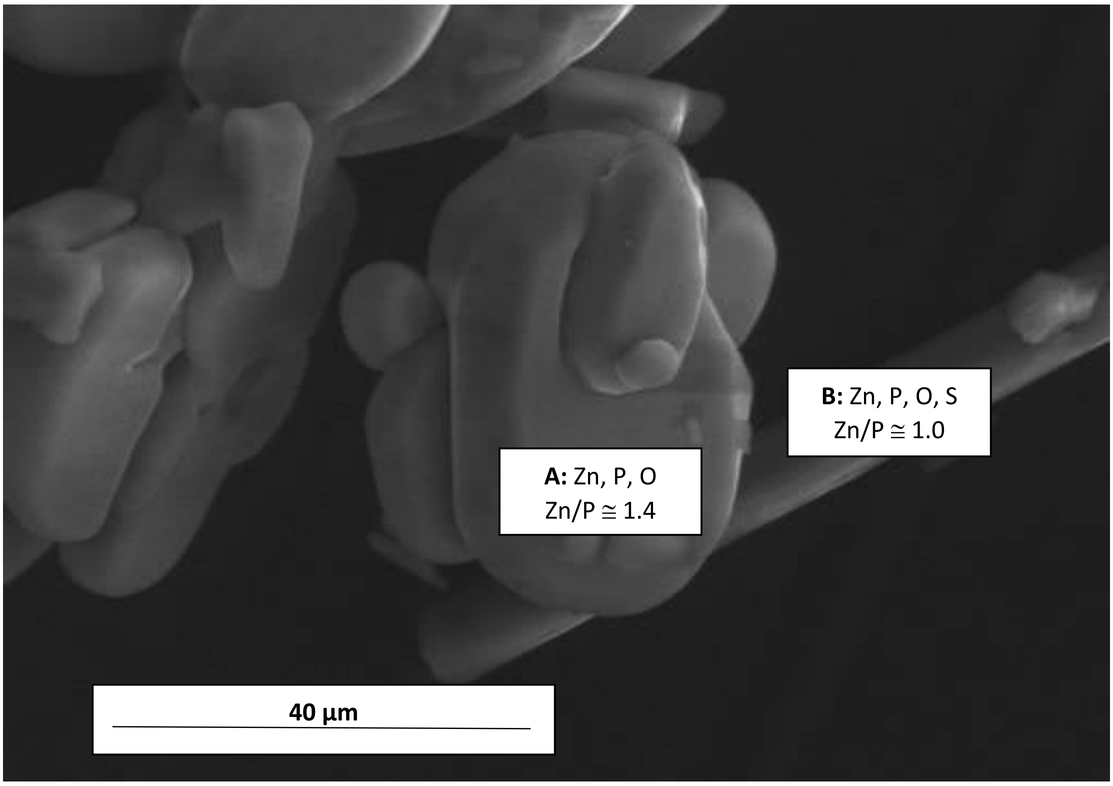

Figure S1. Scanning electron micrograph and semi-quantitative EDS data showing the occurrence of two main types of crystals with different morphology and composition.
Type A crystals are short and thick and contain Zn, P and O, with a Zn/P ~ 1.4 molar ratio.
Type B crystals are long and thinner and contain Zn, P, O and S, with a Zn/P ~ 1.0 molar ratio.
Based on these data, the two types of crystals can be unambiguously assigned to hopeite and (I), respectively.

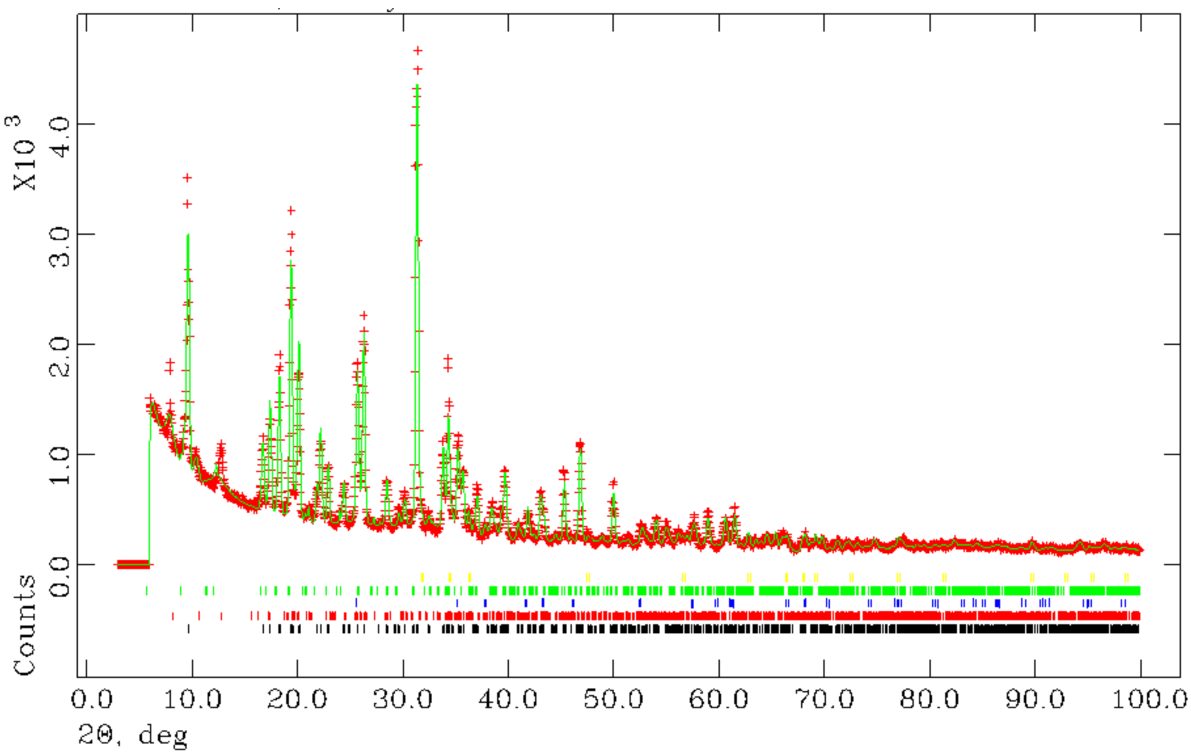

Figure S2. Rietveld refinement plot: experimental (red crosses) and calculated (green line) patterns. Vertical bars indicate the positions of the Bragg reflections for hopeite (black), (I) (red), *L*-methionine (green), zinc oxide (yellow) and α-alumina (blue).

References

[1] PCPFWIN 2.3. JCPDS International center for diffraction data, 637 Swarthmore 2002.
[2] H. M. Rietveld, Acta Crystallogr. 22 (1967) 151–152.
[3] A. Larson, R. Von Dreele, General structure analysis system (GSAS), 1994.
[4] B. Toby, EXPGUI, a graphical user interface for GSAS. J. Appl. Crystallogr. 34 (2001) 210-213.
[5] Inorganic Crystal Structure Database (ICSD), Version 1.9.4, 2014
